# A Unique Case of Mantle Cell Lymphoma Masquerading as a Cecal Mass

**DOI:** 10.1155/2021/5581043

**Published:** 2021-09-10

**Authors:** Sarahi Herrera-Gonzalez, Dema Shamoon, Tingliang Shen, Simon Badin, Yatinder Bains

**Affiliations:** ^1^Department of Internal Medicine, Jersey City Medical Center, 355 Grand St, Jersey City, NJ 07002, USA; ^2^Department of Gastroenterology, St Michael's Medical Center, 111 Central Ave, Newark, NJ 07102, USA; ^3^Department of Pathology, Jersey City Medical Center, 355 Grand St, Jersey City, NJ 07002, USA; ^4^Department of Oncology, Jersey City Medical Center, 355 Grand St, Jersey City, NJ 07002, USA

## Abstract

Mantle cell lymphoma (MCL), a type of B-cell non-Hodgkin's lymphoma, is a rare and aggressive disease with a poor prognosis due to its advanced presentation at diagnosis. It is characterized by a translocation in the Bcl-1 gene, which results in overexpression of cyclin D1. MCL is frequently seen in the form of multiple lymphomatous polyposis (MLP) in which innumerable polyps are observed in the gastrointestinal (GI) tract. In rare instances, MCL presents a single mass. The most common presentation involves male patients in their sixties, with generalized lymphadenopathy, extranodal involvement, and B symptoms (night sweats, fever, and weight loss). Endoscopic findings of MLP include cerebroid folding of the gastric mucosa and innumerable polyps extending from the duodenum to the large intestine and are reported in approximately 9% of all GI lymphomas. Less commonly, only 2–4% of GI malignancies present as a primary GI MCL as a single mass, usually in the stomach and ileocecal region in the intestine. Radiologic findings include lymphadenopathy, splenomegaly, multiple polyposis, or wall thickening with ulceration or mass formation. In most instances, advanced disease is found at diagnosis, for which 5-year survival ranges only from 26 to 46%, even when appropriate treatment is initiated. High mitotic rate, or Ki-67 index, is of prognostic value and is associated with poor prognosis. Treatment involves conventional chemo-immunotherapy consisting of R CHOP (rituximab, cyclophosphamide, doxorubicin, vincristine, and prednisone) or RB (rituximab and bendamustine), with the latter being better tolerated and associated with longer progression-free survival. Surgical resection is usually limited to patients in which complications are seen such as bleeding, perforation, or bowel obstruction. We present a unique case of a 70-year-old male with nonbilious, nonbloody emesis, and symptomatic anemia who was found to have a cecal mass consistent with MCL.

## 1. Introduction

Mantle cell lymphoma (MCL), a type of B-cell non-Hodgkin's lymphoma, is a rare and aggressive disease with a poor prognosis due to its advanced presentation at diagnosis. It is characterized by a translocation in the Bcl-1 gene, which results in overexpression of cyclin D1 [1]. MCL is frequently seen in the form of multiple lymphomatous polyposis (MLP) in which innumerable polyps are observed in the gastrointestinal (GI) tract. In rare instances, MCL presents a single mass often within the gastric mucosa [2]. Chung et al. reported 7 cases of MCL, of which 6 presented with MLP and only one with a single mass which was located in the stomach [3]. Current literature reports GI tract lymphomas as MLP in 9% of cases and less than 4% as a single mass [4]. They are most commonly located in the stomach followed by the ileocecal region [4]. We present a unique case of a patient with emesis and symptomatic anemia who was found to have a cecal mass consistent with MCL.

## 2. Case Presentation

This is a case of a 70-year-old Caucasian male with a medical history of hypertension and osteoarthritis who presented with a two-week history of dizziness. He also reported a few episodes of nonbloody, nonbilious emesis. Four days prior to presentation, he was unable to carry on with his daily activities due to generalized weakness which prompted him to seek medical attention. Upon further review of systems, he was admitted with night sweats, subjective fevers, and loss of appetite resulting in a 40-pound weight loss over the past few months.

On physical exam, the patient had skin and conjunctival pallor. Vitals revealed orthostatic hypotension. Laboratory investigations indicated significant microcytic anemia with a hemoglobin of 7.1 g/dL (14–18 g/dL), decreased from his baseline of 14.5 g/dL one year prior, and a MCV of 66 fL (79–93.9 fL). He had leukocytosis with a white blood cell count of 15.7 (4.5–11.0 K/UL) as well has thrombocytosis of 529 K/UL (130–400 K/UL). On chemistry, he was found to have acute kidney injury with a creatinine level of 1.42 mg/dL (0.7–1.3 mg/dL) and BUN of 34 mg/dL (9–23 mg/dL) from a baseline creatinine level of 0.9 mg/dL. He was also found to have hypokalemia with a potassium level of 3.2 mmol/L (3.5–5.1 mmol/L) and metabolic alkalosis with an elevated CO_2_ level of 34 mg/dL (9–23 mg/dL). Analysis of laboratory values (AKI, mildly elevated leukocytosis, metabolic alkalosis, and hypokalemia) hinted towards dehydration likely resulting volume depletion from vomiting. Laboratory values resolved with hydration. In addition, the patient had a positive stool guaiac suggestive of GI bleed. He was transfused with one unit of packed red blood cells. He denied recent history of hematemesis, melena, or hematochezia and had an unremarkable colonoscopy 5 years prior to presentation.

**Figure 1:**
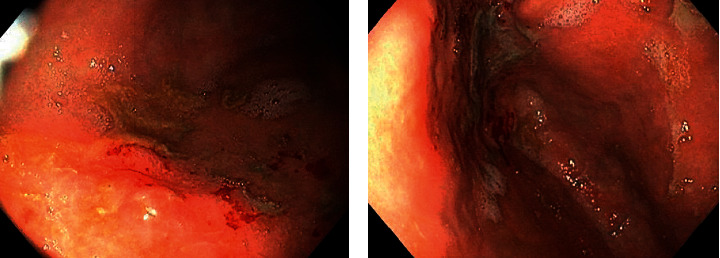
EGD consistent with severe, diffuse hemorrhagic gastritis.

**Figure 2:**
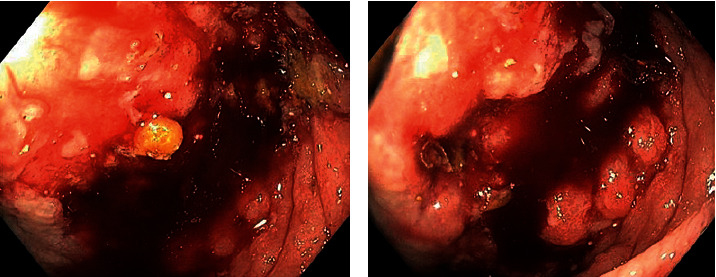
Severe colitis localized at the cecum with friable mucosa.

**Figure 3:**
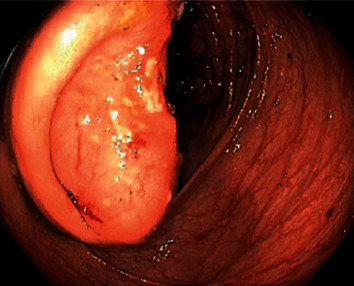
Extraluminal compression and protruding ileocecal valve resulting in narrowing of cecal lumen.

Due to symptomatic anemia, weight loss, and obstructive symptoms in the setting of a GI bleed, he underwent esophagogastroduodenoscopy (EGD) and colonoscopy. EGD revealed severe diffuse hemorrhagic gastritis (Figure 1) with biopsies consistent with chronic gastritis and no malignant features. Colonoscopy showed localized, severe colitis at the cecum that was erythematous, friable, and ulcerative (Figure 2). There was also extraluminal compression of the cecum with protrusion of the ileocecal valve and luminal narrowing (Figure 3).

**Figure 4:**
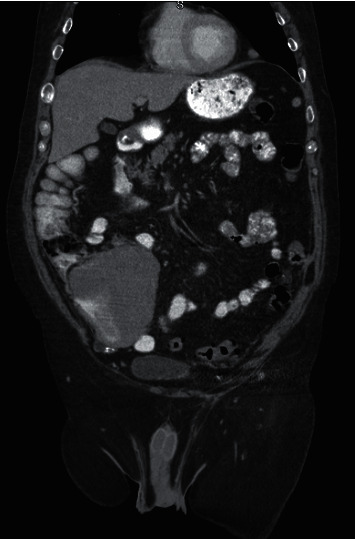
Circumferential cecal mass resulting in narrowing of the terminal ileum and cecal lumen.

Due to the extraluminal compression of the cecum, computed tomography (CT) of the abdomen and pelvis with intravenous and oral contrast was obtained. This ultimately revealed a cecal mass measuring 12 × 12 × 4.7 cm with the bulk of the mass protruding into the terminal ileum causing high-grade narrowing of the lumen (Figure 4). Lymphadenopathy was localized to the right lower quadrant, and no distant lymphadenopathy was observed elsewhere (Figure 4). CT of the chest (with and without IV contrast) that was also done did not show pulmonary nodules, pleural or pericardial effusions, or lymphadenopathy to suggest distant disease.

**Figure 5:**
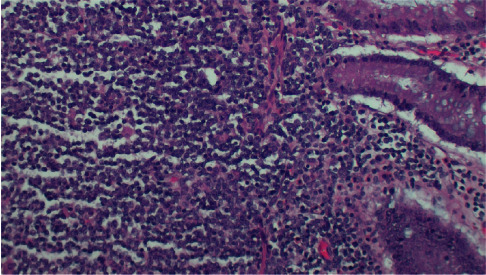
H&E stain showing normal colonic mucosa on the right and small lymphoma cells on left. Cyclin D1+, CD5+ (weak), K67∼68%.

**Figure 6:**
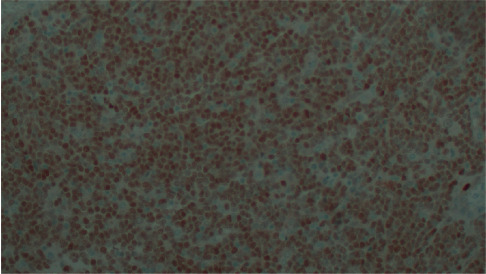
Immunostaining for bcl-1 (cyclin D1).

Subsequently, surgery was consulted, and due to his presentation with obstructive symptoms and symptomatic anemia secondary to the seen cecal mass, the patient underwent a right hemicolectomy. Pathology from both colonoscopy and right hemicolectomy showed MCL of cecum. The lymphoma cells expressed cyclin D, with characteristic cytogenetic abnormality of t (11; 14) (q13; q32). A high index of Ki-67 of 65% indicates adverse prognosis (Figures 5 and 6). MCL was also present in pericolonic lymph nodes.

Initially, the patient declined further workup and treatment including chemotherapy and bone marrow biopsy. Nonetheless, two months after discharge, he presented to the Oncology Clinic willing to undergo further evaluation and treatment. At this time, PET/CT scan revealed advanced disease with hypermetabolic lymph nodes within the mediastinum and retroperitoneum, as well as diffuse omental caking and bilateral malignant pleural effusions; no uptake on the bony structures was identified. The patient was now diagnosed with advanced disease and stage IV MCL, contrary to initial presentation where lymphadenopathy was limited to the right lower quadrant. The patient was started on chemotherapy consisting of rituximab and bendamustine of which he received 2 rounds before being lost to follow-up again.

## 3. Discussion

MCL carries a poor prognosis and represents only 7% of all NHL [5]. Among non-Hodgkin's lymphomas, MCL occurs in a variety of sites from nodal to extranodal locations, with 15 to 30% of patients involving the GI tract [1]. MLP, in which endoscopic findings include cerebroid folding of the gastric mucosa and innumerable polyps extending from the duodenum to the large intestine, is reported in approximately 9% of all GI lymphomas [2, 6]. Moreover, as seen in our patient, only 2–4% of GI malignancies present as a primary GI MCL as a single mass, usually in the stomach and ileocecal region in the intestine [4, 7].

Because histopathological findings of small to medium-sized cells without lymphoepithelial lesions and sparing of germinal center are nonspecific, differentiation between MCL and mucosa-associated lymphoid tissue (MALT) lymphoma requires immunohistochemistry stains [2, 8]. MCL is characterized by a translocation of the Bcl-1 gene t(11; 14) (q13; q32), which is responsible for upregulating cyclin D1 expression. This plays an important role in cell proliferation and is the identifying hallmark of MCL [9]. MCL is also positive for CD5, CD 19, CD20, and CD 22, given their B-cell origin [10].

The most common presentation involves male patients in their sixties, with generalized lymphadenopathy, extranodal involvement, and B symptoms (night sweats, fever, and weight loss) [11]. Our patient presented with weight loss and symptomatic anemia secondary to GI bleed with obstructive symptoms (nausea and vomiting) due to extraluminal compression of the cecum. The mass demonstrated by colonoscopy and CT imaging is a rare finding for lymphoma of the GI tract, thus mimicking the most common GI pathology, adenocarcinoma. To our knowledge, after the report of Assi et al., we are the second reported case of MCL presenting as a single mass causing obstruction [5].

Radiologic findings include lymphadenopathy, splenomegaly, multiple polyposis, or wall thickening with ulceration or mass formation [3]. Extra-abdominal and extranodal involvement has been reported most commonly in the bone marrow, oropharynx, peripheral blood, pleural, mediastinal, breast, and inguinal region [12]. As seen in our patient's initial presentation, a single ileocecal mass with lymphadenopathy limited to the right lower quadrant and without distant involvement was identified. Unfortunately, due to poor follow-up and lack of treatment after surgical intervention, the disease advanced to stage IV metastatic disease.

In most instances, advanced disease is found at diagnosis, for which 5-year survival ranges only from 26–46%, even when appropriate treatment is initiated [12]. High mitotic rate, or Ki-67 index, is of prognostic value and is associated with poor prognosis [7].

Treatment options are based on the patient's overall functional status and age. As a general rule, younger and more fit patients are managed with cytarabine-containing regimens such as R-hyper-CVAD (rituximab plus fractionated cyclophosphamide, vincristine, doxorubicin, and dexamethasone plus methotrexate and cytarabine), R-DHAP (rituximab, dexamethasone, cytarabine, and cisplatin), and R-HAD (rituximab, high-dose cytarabine, and dexamethasone) with or without autologous stem cell transplant (ASCT) consolidation [13, 14]. However, older, less fit patients are treated with conventional chemo-immunotherapy consisting of R CHOP (rituximab, cyclophosphamide, doxorubicin, vincristine, and prednisone) or RB (rituximab and bendamustine), with the latter being better tolerated and associated with longer progression-free survival [4, 11–13]. Surgical resection is usually limited to patients in which complications such as bleeding, perforation, or bowel obstruction are seen on presentation, as seen in our patient [12]. In patients that did not have a partial bowel obstruction or GI bleed, chemotherapy is sufficient for treatment. It is important to obtain biopsies prior to surgery in the absence of the complications listed above to avoid unnecessary surgical intervention.

## 4. Conclusion

We present an interesting case of MCL presenting as bowel obstruction and symptomatic anemia with an incidental cecal mass diagnosed on CT, and colitis and extraluminal narrowing and a protruding ileocecal valve were seen colonoscopy. It is important to be aware of the differentials as management differs which may save a patient from unwarranted surgical intervention.
